# Imaging object-scene relations processing in visible and invisible natural scenes

**DOI:** 10.1038/s41598-019-38654-z

**Published:** 2019-03-14

**Authors:** Nathan Faivre, Julien Dubois, Naama Schwartz, Liad Mudrik

**Affiliations:** 1 Division of Biology, California Institute of Technology, Pasadena, CA 91125 USA; 2 Laboratory of Cognitive Neuroscience, Brain Mind Institute, Faculty of Life Sciences, Swiss Federal Institute of Technology (EPFL), Geneva, Switzerland; 3 Centre d’Economie de la Sorbonne, CNRS UMR 8174 Paris, France; 4 Division of the Humanities and Social Sciences, California Institute of Technology, Pasadena, CA USA; 5 Department of Neurosurgery, Cedars Sinai Medical Center, Los Angeles, CA USA; 6 School of Psychological sciences, Tel Aviv University, Tel Aviv, Israel; 7 Sagol school of Neuroscience, Tel Aviv University, Tel Aviv, Israel

## Abstract

Integrating objects with their context is a key step in interpreting complex visual scenes. Here, we used functional Magnetic Resonance Imaging (fMRI) while participants viewed visual scenes depicting a person performing an action with an object that was either congruent or incongruent with the scene. Univariate and multivariate analyses revealed different activity for congruent vs. incongruent scenes in the lateral occipital complex, inferior temporal cortex, parahippocampal cortex, and prefrontal cortex. Importantly, and in contrast to previous studies, these activations could not be explained by task-induced conflict. A secondary goal of this study was to examine whether processing of object-context relations could occur in the absence of awareness. We found no evidence for brain activity differentiating between congruent and incongruent invisible masked scenes, which might reflect a genuine lack of activation, or stem from the limitations of our study. Overall, our results provide novel support for the roles of parahippocampal cortex and frontal areas in conscious processing of object-context relations, which cannot be explained by either low-level differences or task demands. Yet they further suggest that brain activity is decreased by visual masking to the point of becoming undetectable with our fMRI protocol.

## Introduction

A very short glimpse of a visual scene often suffices to identify objects, and understand their relations with one another, as well as with the context in which they appear. The observed co-occurrences of objects and their relations within specific scenes or contexts^1^ shape our expectations. When these expectations are violated^2^, object and scene processing are impaired - both with respect to speed^3–5^ and to accuracy ^6–8^, suggesting that contextual expectations may have an important role in scene and object processing^1,9,10^, though see^11^.

Yet the mechanisms of contextual facilitation for object/scene comprehension are still unclear. Several studies have tried to track the neural substrates of contextual processing and gist extraction^10^, suggesting parallel yet interacting streams for object and scene processing^12^, and emphasizing the role of expectations in visual perception^13^. Specifically for processing the relations between objects and scenes, an interplay between frontal and temporal visual areas has been suggested^1^(see also^13–15^), so that after identifying the scene’s gist, high-level contextual expectations about scene-congruent objects are formed. The activated representations of such objects are compared with upcoming visual information about objects’ features, until a match is found and the objects are identified (for electrophysiological support, see^9,16–19^,though see^20^).

However, these suggestions are mostly based on studies that did not directly examine the processing of objects embedded in scenes, but rather used other ways to probe contextual processing (e.g., comparing objects that evoke strong vs. weak contextual associations^21^, or manipulating the relations between two isolated objects^22,23^). Critically, the few papers that did focus on objects embedded in real life scenes^24–26^ report conflicting findings about the role of frontotemporal regions - more specifically the prefrontal cortex (PFC) and the medial temporal lobe (MTL). Several areas within the prefrontal cortex have been implicated in semantic processing (e.g., left Inferior PFC^27^; bilateral Inferior PFC, bilateral Superior Frontal Gyrus, right Middle Frontal Sulcus, Cingulate^22^; medial PFC^21,28^), including a direct manipulation of object-scene relations (right Middle Frontal Sulcus and Inferior PFC^24^). However, it has been suggested that these frontal activations may stem from task-induced conflict rather than from the actual processing of object-context relations^24^.

Likewise, the literature is divided about the involvement of the MTL in such processing. In particular, for the parahippocampal cortex (PHC), some have suggested a functional dissociation^29^ whereby anterior parts process contextual associations while posterior parts process spatial layouts^24,30–32^ (though Rémy and colleagues reported semantic effects on both posterior and anterior PHC; see also^33^, who reported PHC and hippocampal activations for contextual binding between objects and scenes, and^34^ that gave an account of PHC activity in terms of statistical learning of object co-occurrences). Others argue that the PHC is solely dedicated to processing spatial layouts (the spatial layout hypothesis^35,36^; see also^37^ and^38^ that support the spatial account, yet interestingly find effects of both the scene and its constituents on PHC activity), or representations of three-dimensional local spaces, even of a single object^39^, irrespective of contextual associations^40^.

Other regions participating in scene processing have also been suggested: namely, the retrosplenial cortex (RSC) and Occipital Place Area (OPA) were both implicated in scene perception and in contextual processing^29^. Yet RSC involvement in scene processing is commonly held to be related to navigation^41,42^, without being affected by the objects in the scene^38^. And OPA activity is considered to reflect more perceptual-level processing of features characteristic of scenes^43^. Thus, it is not clear whether these areas should also be involved in processing object-scene relations.

 As outlined above, the neural mechanisms underlying the processing of object-context relations are still under investigation; and so are the cognitive characteristics of this phenomenon. For instance, whether conscious perception is necessary for the processing of object-context relations is still unresolved. Using behavioral measures, two previous studies suggested that integration of object and scene can occur even when subjects are unaware of both objects and the scenes in which they appear^44,45^ (for prioritized access to awareness of interacting vs. non-interacting objects, see^46^). This result is in line with the *unconscious binding hypothesis*^47^, according to which the brain can associate, group or bind certain features in invisible scenes, especially when these features are dominant (for a discussion of conscious vs. unconscious integration, see^48^). However, two recent attempts to replicate these findings have failed^49,50^. This absence of an effect accords with theories that tie integration with consciousness (Global Workspace Theory^51,52^; Integrated Information Theory^53,54^). While the jury is still out on this question, our study aimed at measuring the brain activity mediating the processing of unconscious object-context relations - if indeed such processing is possible in the absence of awareness.

**Figure 1:**
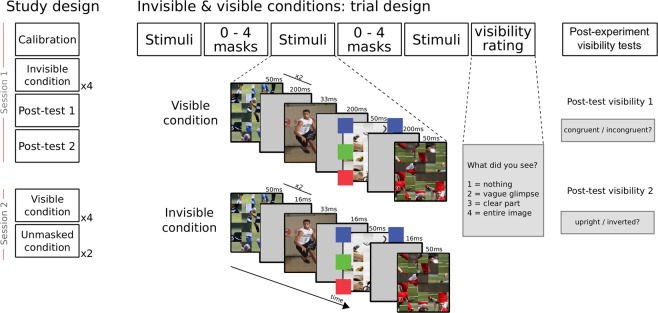
Experimental procedure. The left column depicts the different sessions and their order (calibration, invisible condition, visibility tests, visible condition and unmasked condition). At the center, the experimental sequence in the main runs, and on the right are the two post-test visibility runs. In all trials, one experimental sequence was repeated three times, separated by 0–4 masks to avoid temporal predictability of the target image. The experimental trials ended with only one question about target visibility. In the post-test trials, this subjective visibility question was preceded by an objective question (about target congruency/orientation). Each experimental sequence included one presentation of the target scene, which was either congruent or incongruent, and forward and backwards two masks which either immediately followed and preceded the scene (invisible condition) or were separated from it by blank frames (visible condition). Thus, throughout each trial, the target scene was presented three times.

The goals of the current study were thus twofold; first, we aimed at identifying the neural substrates of the processing of object-context relationships, and specifically at testing whether frontal activations indeed reflect contextual processing rather than task-related conflict^24^. Second, we looked for evidence of unconscious processing of object-context relationships while carefully controlling visibility, with the goal of identifying the neural substrates of such processing in the absence of awareness. Subjects were thus scanned as they were presented with masked visual scenes depicting a person performing an action with a congruent (e.g., a man drinking from a bottle) or an incongruent (e.g., a man drinking from a flashlight) object. The experiment had two conditions: one in which scenes were clearly visible (visible condition), and one in which they were not (invisible condition) (see Fig. 1). Stimulus visibility was manipulated by changing masking parameters while keeping stimulus energy constant. Participants rated stimulus visibility on each trial; they did not perform any object-context congruency or object-identification judgments, to ensure that the measured brain activations could be attributed to object-context integration per se, and not to task-induced conflict^24^.

## Methods

### Participants

Eighteen participants (eight females, mean age = 25.1 years, SD = 4.32 years) from the student population of the California Institute of Technology took part in this study for payment ($50 per hour). All participants were right-handed, with normal or corrected-to-normal vision, and no psychiatric or neurological history. The experiment was approved by the Institutional Review Board committee of the California Institute of Technology, and all methods were performed in accordance with the corresponding guidelines and regulations. Informed consent was obtained after the experimental procedures were explained to the subjects. Two additional participants had too few invisible trials in the invisible condition (less than 70% of trials), and were excluded from the analysis.

### Apparatus and stimuli

Stimuli were back-projected onto a screen that was visible to subjects via a mirror attached to the head coil, using a video projector (refresh rate 60 Hz, resolution 1280 × 1024). Stimuli were controlled from a PC computer running Matlab with the Psychophysics Toolbox version 3^55–57^. Responses were collected with two fiber optic response devices (Current Designs, Philadelphia, USA). Target images were 6.38° by 4.63° (369 × 268 pixels) color pictures of a person performing an action with an object. In the congruent condition, the object was congruent with the performed action (e.g., a woman drinking from a cup), while in the incongruent condition, it was not (e.g., a woman drinking from a plant; see^45,58^ for details). In both types of images, the critical object was pasted onto the scene. Low-level differences in saliency, chromaticity, and spatial frequency were controlled for during the creation of the stimulus set^9^, and tested using an objective perceptual model^59^. Visual masks were generated from a different set of scenes, by cutting each scene image into 5 × 6 tiles and then randomly shuffling the tiles.

### Procedure

The experiment was run over two separate one-hour scanning sessions on two consecutive days. The first session included a *Calibration* condition (which was conducted during the anatomical scan), four runs of the *Invisible* condition, and two post-experiment visibility tests. The second session included four runs of the *Visible* condition, followed by two runs of an *Unmasked* condition in which scenes were presented in blocks for longer durations, and with no masks (Fig. 1). Note that the visible and unmasked conditions were conducted in the second session, so the results in the invisible condition would not be biased by previous conscious exposure. Thus, the visible condition was aimed at achieving the first goal of this research, which was to identify the neural activations underlying object-scene relations processing. The unmasked condition was added in case the mere presence of masks in the visible condition hindered such processing. The invisible condition was aimed at achieving the second goal of this research – to look for neural evidence for object-scene relations processing in the absence of awareness.

#### Calibration condition

The calibration was designed to individually adjust the contrasts of the masks and targets, thus ensuring a comparable depth of suppression across subjects in the invisible condition. 72 images (half congruent, half incongruent; all different from the ones used in the main experiment) were presented, either upright or inverted (pseudo-randomly intermixed, with the constraint that the same image orientation was never presented in four consecutive trials). Subjects indicated for each trial whether the image was upright or inverted, and rated its visibility subjectively using the Perceptual Awareness Scale (PAS^60^), where 1 signifies “I didn’t see anything,” 2 stands for “I had a vague perception of something,” 3 represents “I saw a clear part of the image,” and 4 is “I saw the entire image clearly.” Subjects were instructed to guess the orientation if they did not see the image. Initial mask (Michelson) contrast was 0.85, and initial prime contrast was 0.7. Following correct responses (i.e., correct classification of the image orientation as upright or inverted), mask contrast was increased by 0.05, and following incorrect responses, it was decreased by 0.05 (i.e., 1-up, 1-down staircase procedure^61^). If mask contrast reached 1, target contrast was decreased by steps of 0.05, stopping at the minimum allowable contrast of 0.15. In the main experiment, mask contrast was then set to the second highest level reached during the calibration session (i.e., if the highest mask contrast during the calibration reached a value of 0.95, it was set to 0.90). If mask contrast reached 1, it was set to 1, and target contrast was then set to the second lowest level reached (i.e., if it reached a value of 0.4, it was set to 0.45). Mask contrast reached 1 for all subjects. Average target contrast was 0.39 ± 0.05 (here and elsewhere, ± denotes 95% confidence interval). The same contrasts were used in both visible and invisible conditions in the main experiment, so stimulus energy entering the system would be matched.

#### Main experimental conditions

The invisible and visible conditions were each divided into four runs of 90 trials, of which 72 contained either congruent or incongruent target images, and 18 had no stimuli (i.e., “catch trials”), serving as baseline. Thus, since we had 144 pairs of images, each image repeated two times in each condition. The order of congruent and incongruent trials within a run was optimized using a genetic algorithm^62^ with the constraint that a run could not start with a baseline trial, and that two baseline trials could not occur in succession. Each trial started with a fixation cross presented for 200 ms, followed by three repeats of a sequence of target images and masks (the sequence was repeated three times to allow for better processing of the masked scene and increase its signal^63^), and then a judgment of image visibility using the PAS. The sequence started with two forward masks (each presented for 50 ms, with a 17 ms blank interval), followed by the target image (33 ms), and two backward masks (50 ms each, 17 ms blank interval). The only difference between the invisible condition (first session) and the visible condition (second session) lied in the duration of the intervals immediately preceding and immediately following the target image: a 17 ms gap was used in the invisible condition, while a 50 to 200 ms gap (randomly selected on each trial from a uniform distribution) was used in the visible condition. To equate the overall energy of a trial across conditions, the final fixation in the sequence lasted between 100 and 400 ms in the invisible condition. A random number of masks (0–4) was presented between repetitions of the sequence in each trial, to minimize predictability of the onset of the target image (Fig. 1). A random inter-trial interval (uniform distribution between 1 and 3 s) was enforced between trials, so that on average a whole trial lasted 4.5 s.

#### Post-experiment visibility tests

At the end of the first session, following the invisible condition, two objective performance tasks were administered to behaviorally validate that the masking procedure was effective in suppressing the scenes from awareness. First a congruency task performed in the scanner, in which subjects were asked to determine if a scene was congruent or not (2-AFC, 90 trials). Second, an orientation task performed outside the scanner, in which half the images were upright and half were inverted, and subjects were asked to determine their orientation (2-AFC, 72 trials). In both tasks, the same trial structure and the same images as the ones used in the main experiment were used; subjects were instructed to guess if they did not know the answer. Subjects also rated image visibility using the PAS, after each trial.

#### Unmasked condition

At the end of the second session, following the visible condition, subjects participated in two runs of a block design paradigm to localize brain regions that respond differentially to congruent and incongruent scenes, in the absence of visual masks. This session served as an alternative to the visible condition, in case the short presentation of the stimuli and the presence of masks might evoke too weak responses. Each run consisted of 18 blocks of 12 images, which were either all congruent or all incongruent scenes – the same scenes which were presented in the main experimental conditions. Blocks started with a 5–7 s fixation cross. Then, the 12 images were presented successively for 830 ms each, with a 190 ms blank between images. Subjects had to detect when an image was repeated, which occurred once per block (1-back task). When analyzing the data, we verified that regions responding differently to congruent and incongruent scenes were consistent across the unmasked and the visible conditions.

### Behavioral data analysis

All analyses were performed with R (2016), using the BayesFactor^64^, and ggplot2^65^ packages.

### MRI data acquisition and preprocessing

All images were acquired using a 3 Tesla whole-body MRI system (Magnetom Tim Trio, Siemens Medical Solutions) with a 32-channel head receive array, at the Caltech Brain Imaging Center. Functional blood oxygen level dependent (BOLD) images were acquired with T2*-weighted gradient-echo echo-planar imaging (EPI) (TR/TE = 2500/30 ms, flip angle = 80°, 3 mm isotropic voxels and 46 slices acquired in an interleaved fashion, covering the whole brain). Anatomical reference images were acquired using a high-resolution T1-weighted sequence (MPRAGE, TR/TE/TI = 1500/2.74/800 ms, flip angle = 10°, 1 mm isotropic voxels).

The functional images were processed using the SPM8 toolbox (Wellcome Department of Cognitive Neurology, London, UK) for Matlab. The first three volumes of each run were discarded to eliminate nonequilibrium effects of magnetization. Preprocessing steps included temporal high-pass filtering (1/128 Hz), rigid-body motion correction and slice-timing correction (middle reference slice). Functional images were co-registered to the subject’s own T1-weighted anatomical image. The T1-weighted anatomical image was segmented into gray matter, white matter and cerebrospinal fluid, and nonlinearly registered to the standard Montreal Neurological Institute space distributed with SPM8 (MNI152). The same spatial normalization parameters were applied to the functional images, followed by spatial smoothing (using a Gaussian kernel with 12 mm full-width at half maximum) for group analysis. Scans with large signal variations (i.e., more than 1.5% difference from the mean global intensity) or scans with more than 0.5 mm/TR scan-to-scan motion were repaired by interpolating the nearest non-repaired scans using the ArtRepair toolbox^66^.

### fMRI analysis

Statistical analyses relied on the classical general linear model (GLM) framework. For univariate analyses, models included one regressor for congruent, one regressor for incongruent, and one regressor for baseline trials in each run. Each regressor consisted in delta functions corresponding to trial onset times, convolved with the double gamma canonical hemodynamic response function (HRF). Time and dispersion derivatives were added to account for variability of the HRF across brain areas and subjects. Motion parameters from the rigid-body realignment were added as covariates of no interest (6 regressors). Individual-level analyses investigated the contrast between the congruent vs. the incongruent condition. Group-level statistics were derived by submitting individual contrasts to a one sample two-tailed t-test. We adopted a cluster-level thresholding with p < 0.05 after FWE correction, or uncorrected threshold with p < 0.001 and a minimal cluster extent of 10 voxels. Note that our results did not resist correction for multiple comparisons after threshold free cluster enhancement (TFCE^67^). The unmasked session followed the same preprocessing and analysis steps as the main experimental runs. For multivariate analyses, each run was subdivided into four mini-runs, each containing nine congruent and nine incongruent trials. One regressor was defined for congruent and incongruent trials in each mini-run. No time or dispersion derivative was used for these models, and no spatial smoothing was applied. The individual beta estimates of each mini-run were used for classification. Beta estimates of each voxel within a given region of interest (ROI) were extracted and used to train a linear Support Vector Machine (using the libsvm toolbox for Matlab, http://www.csie.ntu.edu.tw/cjlin/libsvm) to classify mini-runs into those with congruent and incongruent object-context relations. A leave-one-run-out cross-validation scheme was used. Statistical significance of group-level classification performances was assessed using Bayes Factor and permutation-based information prevalence inference to compare global vs. majority null hypotheses^68^. ROIs were defined based on the contrast in the unmasked condition, by centering spheres of 12 mm radius on the peak activity of each cluster of more than five contiguous voxels (voxel-wise threshold p < 0.001, uncorrected). Note that the inter-individual variability did not allow defining ROIs at the individual level.

## Results

### Behavioral data

**Figure 2:**
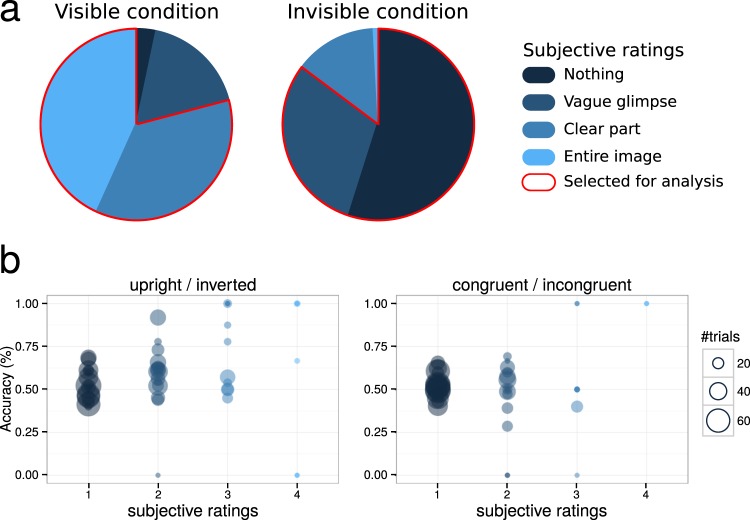
Behavioral results. (**a**) Average distributions of subjective ratings in the visible and invisible conditions. In the visible condition, only trials in which participants reported seeing a clear part or the entire image were selected for analysis. In the invisible condition, these trials were excluded, while those in which nothing or a vague glimpse was perceived were kept. (**b**) Objective performance for discriminating upright/inverted scenes (left panel) and congruent/incongruent scenes (right panel) in the invisible condition. Each circle represents individual performance in one category of subjective visibility. The size of each circle represents the number of trials from which individual performance was computed.

In the visible condition, subjects’ visibility ratings indicated that target images were partly or clearly perceived (percentage of total trials: visibility 1: 3.5% ± 3.0%; visibility 2: 18.5% ± 7.5%; visibility 3: 37.8% ± 5.4%; visibility 4: 45.4% ± 9.4%). Only trials with visibility ratings of 3 or 4 were kept for further analyses. By contrast, in the invisible condition, subjective visibility of target images was dramatically reduced due to masking (visibility 1: 56.6% ± 12.2%; visibility 2: 31.2% ± 7.1%; visibility 3: 14.3% ± 7.8%; visibility 4: 0.1% ± 0.04%) (Fig. 2a). Only trials with visibility ratings of 1 or 2 were kept for further analyses (for a similar approach, see^69^). Objective performance for discriminating scene congruency in corresponding visibility ratings in the invisible condition did not significantly differ from chance-level (51.6% ± 2.5%, t(15) = 1.24, p = 0.23, BF = 0.49, indicating that H_0_ was two times more likely than H_1_) (Fig. 2b). However, objective performance for discriminating upright vs. inverted scenes was slightly above chance-level (56.0% ± 4.4%, t(16) = 2.64, p = 0.02, BF = 3.3, indicating that H_1_ was around three times more likely than H_0_; regrettably, two subjects did not complete the objective tasks due to technical issues). This suggests some level of partial awareness, in which some participants could discriminate low-level properties of the natural scene such as its vertical orientation, but not its semantic content^70,71^. In all following results, unconscious processing is therefore defined with respect to scene congruency, and not to the target images themselves of which subjects might have been partially aware, at least in some trials.

### Imaging data

#### Univariate analyses

The set of brain regions involved in the processing of scene congruency was first identified using the unmasked condition, contrasting activity elicited by congruent vs. incongruent blocks of target images (no masking, see Methods). At the group level, this contrast revealed a set of regions in which activity was weaker for congruent than incongruent images (here and elsewhere, no activations in the other direction – weaker for incongruent images – were found). Significant activations (for cluster-level thresholding with p < 0.05 after FWE correction, or uncorrected threshold with p < 0.001 and a minimal cluster extent of 10 voxels, for exploratory purposes) in the temporal lobe comprised the right inferior temporal and fusiform gyri extending to the posterior parahippocampal gyrus and the left middle temporal gyrus (see Table 1). In the parietal lobe, activations were detected in the bilateral cunei, the right parietal lobule, and the left paracentral lobule. In the frontal lobe, significant clusters were observed in the bilateral inferior frontal gyri, right middle frontal gyrus, left superior medial frontal gyrus, and left cingulate gyrus.

**Table 1:** Cluster extent (CE), statistical values, and MNI stereotaxic brain atlas coordinates for the brain regions more activated by incongruent vs. congruent target images during the unmasked condition.

Cluster size	T-value (peak)	P-value	correction	x	y	z	Anatomical region	Broadmann area
2365	8.47	<0.001	FWE	57	−61	−11	Right Inferior Temporal Gyrus	BA 37
	8.27		FWE	30	−55	49	Right Precuneus	BA 7
	7.41		FWE	27	−70	40	Right Superior Parietal Lobule	BA 7
1215	7.81	<0.001	FWE	−42	2	34	Left Inferior Frontal Gyrus	BA 9
	6.28		FWE	−27	17	−2	Left Claustrum	
2538	7.72	<0.001	FWE	−33	−58	−11	Left Cerebellum	
	7.11		FWE	−48	−49	−14	Left Sub-Gyral	BA 37
	7.03		FWE	−36	−67	−5	Left Middle Occipital Gyrus	BA 37
347	5.74	0.001	FWE	48	14	34	Right Middle Frontal Gyrus	BA 9
	4.51		FWE	57	35	19	Right Middle Frontal Gyrus	BA 46
143	4.98	0.033	FWE	0	−31	31	Left Cingulate Gyrus	BA 23
	3.88	<0.001	unc	0	−34	46	Left Paracentral Lobule	BA 31
55	4.41	<0.001	unc	−3	20	52	Left Medial Frontal Gyrus	BA 8
	3.77	<0.001	unc	−12	23	67	Left Superior Frontal Gyrus	BA 6
45	4.26	<0.001	unc	24	20	−14	Right Inferior Frontal Gyrus	BA 47
	4.22	<0.001	unc	21	35	−17	Right Middle Frontal Gyrus	BA 11
	4.02	<0.001	unc	39	32	−14	Right Inferior Frontal Gyrus	BA 47
15	4.14	<0.001	unc	9	11	10	Right Caudate	Caudate Body
16	4.11	<0.001	unc	−9	35	28	Left Anterior Cingulate	BA 32
10	4	<0.001	unc	−9	−73	−35	Left Cerebellum	
19	3.93	<0.001	unc	51	38	−2	Right Middle Frontal Gyrus	BA 47

**Figure 3:**
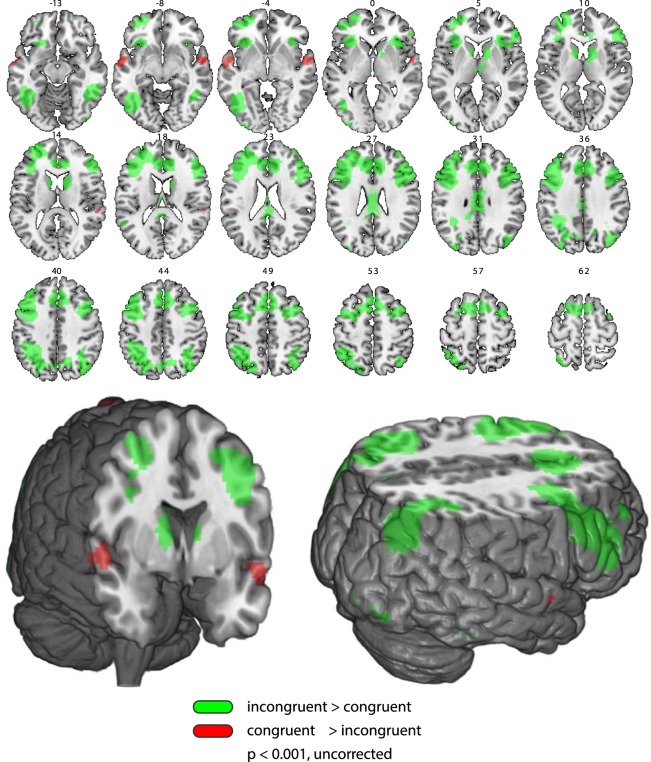
T-maps for the comparison of congruent vs. incongruent images during the visible condition (p < 0.001, uncorrected; see tables for corrected p-values). Regions in red were more active following congruent vs. incongruent images. Regions in green were more active following incongruent vs. congruent images. Activations in regions which were not identified in the unmasked condition included the following areas: in the right hemisphere, the inferior occipital, medial and superior frontal, and orbital gyri. In the left hemisphere – the superior and middle occipital, parahippocampal, fusiform, inferior temporal, supramarginal, and middle frontal gyri, as well as the inferior parietal lobule, posterior cingulate cortex, and caudate body.

We then used results from the unmasked condition as an inclusive mask when contrasting activity elicited by congruent vs. incongruent target images during the visible condition in the main experimental runs (see Fig. 3 and Table 2). Significant activations were found bilaterally in the fusiform (including the posterior parahippocampal gyrus), cingulate, middle and inferior frontal gyri, inferior parietal lobules, precunei, insular cortices, and caudate bodies; as well as activations in the left hemisphere, including the left inferior and superior temporal gyri, and left medial frontal gyrus (see Table 2).

**Table 2:** Cluster extent (CE), statistical values, and MNI stereotaxic brain atlas coordinates for the brain regions more activated by incongruent vs. congruent target images during the main task in the visible condition.

Cluster size	T-value (peak)	P-value	correction	x	y	z	Anatomical region	Broadmann area
4208	7.37	<0.001	FWE	9	29	40	Right Medial Frontal Gyrus	BA 6
	7.28		FWE	−12	32	28	Left Anterior Cingulate	BA 32
	6.87		FWE	39	14	34	Right Middle Frontal Gyrus	BA 9
258	7.01	0.001	FWE	−3	−13	28	Left Cingulate Gyrus	BA 23
	5.92		FWE	−6	−25	31	Left Cingulate Gyrus	BA 23
	5.5		FWE	−3	−40	19	Left Posterior Cingulate	BA 29
143	6.9	0.022	FWE	48	−43	−17	Right Fusiform Gyrus	BA 37
	4.63		FWE	54	−34	−17	Right Inferior Temporal Gyrus	BA 20
827	6.65	<0.001	FWE	15	−64	40	Right Precuneus	BA 7
	6.41		FWE	−33	−49	40	Left Inferior Parietal Lobule	BA 40
	4.58		FWE	−27	−70	40	Left Precuneus	BA 7
344	5.7	<0.001	FWE	−42	−67	−8	Left Inferior Temporal Gyrus	BA 37
	5.49		FWE	−45	−52	−14	Left Fusiform Gyrus	BA 37
143	5.03	0.022	FWE	12	11	10	Right Caudate	Caudate Body
	4.32		FWE	0	−16	4	Left Thalamus	Medial Dorsal Nucleus
	3.98		FWE	9	−7	7	Right Thalamus	Ventral Anterior Nucleus
259	4.97	0.001	FWE	39	−79	34	Right Precuneus	BA 19
	4.9	<0.001	unc	42	−73	40	Right Precuneus	BA 19
	4.78	<0.001	unc	39	−67	46	Right Inferior Parietal Lobule	BA 7
52	4.83	<0.001	unc	−12	8	10	Left Caudate	Caudate Body
29	4.62	<0.001	unc	39	53	25	Right Middle Frontal Gyrus	BA 10
16	4.22	<0.001	unc	−33	−94	1	Left Middle Occipital Gyrus	BA 18
	3.64	<0.001	unc	−39	−91	7	Left Middle Occipital Gyrus	BA 18
10	3.75	<0.001	unc	−60	−49	19	Left Supramarginal Gyrus	BA 40

When contrasting activity elicited by congruent vs. incongruent target images in the invisible condition, no significant result was found with the same uncorrected threshold of p < 0.001. We verified that the images did elicit a response in visual cortices irrespective of their congruency, by contrasting both congruent and incongruent trials with the baseline condition in which no target image was shown (see fig. SI1). Thus, masking was not so strong as to completely prevent low-level visual processing of the target images.

#### Multivariate analyses

**Figure 4:**
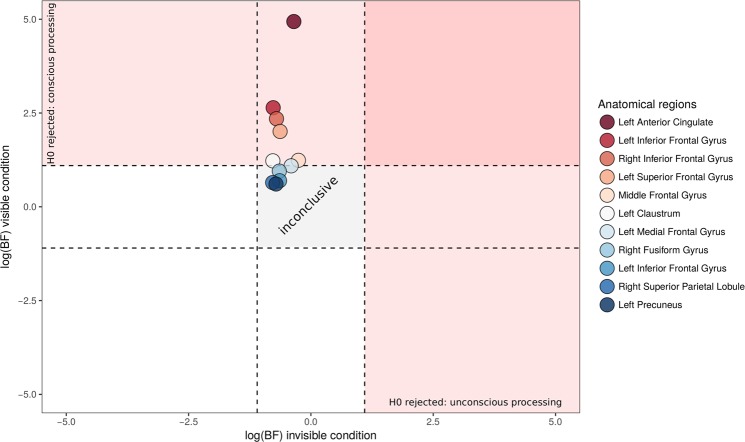
Logarithm of Bayes factor corresponding to decoding performances in the visible (y-axis) and invisible (x-axis) conditions, in the spherical ROIs where significant decoding was found in the visible condition. Dashed lines represent values below which the null hypothesis is favored (horizontal and vertical intercepts of log(1/3)), and above which the null hypothesis can be rejected (horizontal and vertical intercepts of log(3)). Note that all results in the invisible condition lay in the “gray zone” where the null hypothesis can neither be supported or rejected, suggesting inconclusive data; while most areas are above the dashed line in the visible condition, where the null hypothesis is rejected.

Although congruent and incongruent scenes did not elicit different magnitudes of activity in the invisible condition, one possibility is that they elicited different patterns of activity^72^. To test this possibility, we investigated whether the activity patterns extracted from spheres centered on the clusters identified in the unmasked condition conveyed additional information regarding scene congruency. In the visible condition, we could decode scene congruency from the spheres located in the right fusiform gyrus, right superior parietal lobule, middle and inferior frontal gyrus, left precuneus, left superior medial and inferior frontal gyrus, and left anterior cingulate cortex. (Fig. 4, Table 3). In the invisible condition, on the other hand, we could not decode congruency above chance in any of the spherical ROIs, with a statistical significance threshold of p < 0.05 for the global and majority null hypotheses (see Methods). Bayesian analyses revealed that our results in the invisible condition were inconclusive: evidence was lacking in our data to support the null hypothesis (i.e., log(1/3) < log(BF) > log(3), see Fig. 4).

**Table 3:** Average decoding performance, p-values for the global and majority null hypotheses (permutation-based information prevalence inference), logarithm of Bayes factors in the visible condition in the main task, and MNI stereotaxic brain atlas coordinates for the spheres centered on brain regions defined in the unmasked condition.

Decoding performance	Global null p-value	Majority null p-value	log Bayes Factor	x	y	z	Region of Interest	Broadmann area
53.8	0.026	0.14	0.95	48	−55	−17	Right Fusiform Gyrus	BA 37
55.6	0.043	0.73	0.65	27	−70	40	Right Superior Parietal Lobule	BA 7
54.9	0.039	0.3	0.7	−42	2	34	Left Inferior Frontal Gyrus	BA 9
55	0.045	0.74	0.61	−27	−49	46	Left Precuneus	BA 7
55.9	0.017	0.05	1.24	−36	20	25	Middle Frontal Gyrus	BA 9
56.8	0.002	0.05	2.64	−45	8	28	Left Inferior Frontal Gyrus	BA 9
56.8	0.017	0.86	1.23	−27	17	−2	Left Claustrum	
56.8	0.021	0.31	1.09	−3	20	52	Left Medial Frontal Gyrus	BA 8
57.1	0.006	0.31	2.01	−12	23	67	Left Superior Frontal Gyrus	BA 6
54.9	0.003	0.13	2.35	24	20	−14	Right Inferior Frontal Gyrus	BA 47
54.9	0.012	0.13	1.5	39	32	−14	Right Inferior Frontal Gyrus	BA 47
58.7	0.001	0.13	4.94	−9	35	28	Left Anterior Cingulate	BA 32

The majority null hypothesis was rejected at a threshold p < 0.05 only in the left middle and inferior frontal gyri, suggesting that decoding was possible in a majority of participants in these brain regions.

## Discussion

The current study aimed at determining the neural substrates for the processing of object-context relations that are recruited irrespective of a specific task or instruction, and assessing the existence of such processing even when subjects are unaware of the presented scenes. While for the first question an extensive network of areas was identified – overcoming possible previous confounds induced by task demands or low-level features of the stimuli - the data was inconclusive with respect to answering the second question. We were unable to detect any neural activity which differentiated between congruent and incongruent scenes in the invisible condition, though as we explain below, this might stem from limitations of our study.

### Neural correlates of object-context integration

In line with previous studies, several areas emerged when contrasting activations induced by congruent vs. incongruent images. A prominent group of visual areas were identified: the inferior temporal gyrus, suggested by Bar^1^ (see also^15^) as the locus of contextually-guided object recognition, and the fusiform gyrus, previously implicated also in associative processing^73,74^. In line with this finding, the Lateral Occipital Complex (LOC), which comprises the posterior part of the fusiform gyrus, was previously reported to differentiate between associatively related and unrelated objects^22,74,75^. Interestingly, in a recent study which also presented congruent and incongruent scenes^24^, the LOC did not show stronger activity for incongruent images but only responded to animal vs. non-animal objects, irrespective of the scene in which they appeared. Possibly, this discrepancy could be explained by a difference in stimuli. All of our scenes portray a person performing an action with an object, so that object identity is strongly constrained by the scene (the number of possible objects which are congruent with the scene is very small; e.g., a person can only drink from a limited set of objects). In most previous studies, including that of Rémy and colleagues^24^, the scene is a location (e.g., a street, the beach, a living room), in which many objects could potentially appear. Arguably, the stronger manipulation of congruency induced by our stimuli elicited wider activations.

Our results also suggest that the posterior – and not solely the anterior – parahippocampal cortex (PHC), most likely corresponding to the Parahippocampal place area (PPA), is involved in processing contextual associations^22,24,30–32,76,77^, in line with a previous study which also found posterior activations in response to incongruent scenes^24^. Critically, these activations cannot be explained by spatial layouts^35,36,38,39^, since the scenes were identical in that respect, so that the only difference between them is the congruent/incongruent object that was pasted onto them. Furthermore, our study overcomes another, more specific possible confound^24^, which relates to spatial frequencies. The latter were found to modulate PHC activity^78,79^, implying that its object-related activation might be at least partially explained by different spatial properties of these stimuli, rather than by their semantic content. In the current study, all scenes were empirically tested for low-level visual differences between the conditions, including spatial frequencies, and no systematic difference was found between congruent and incongruent images^9^, suggesting that the scenes differ mainly - if not exclusively - in contextual relations between the object and the scene in which it appears. Thus, our results go against low-level accounts of PHC activity, and suggest that it more likely reflects associative processes. This is in line with previous findings of PHC activations in response to objects which entail strong, as opposed to weak, contextual associations^21,31^; thus, in both cases the PHC was recruited when associative processing was evoked, either by objects that entail strong associations or by scenes in which these associations are defied (note again though that our findings were more focused on posterior PHC, like in Rémy *et al*.^24^, which might be explained by the usage of scenes vs. isolated objects).

Finally, our results strengthen the claim that different frontal areas (the inferior frontal gyri^24^, the medial frontal gyrus^28^ (notably, there a very different paradigm was used, and this area showed elevated activity for retrieval of previously learned congruent associations) and the cingulate cortex^22^ are indeed involved in the processing of object-scene relations (in fact, these areas elicited the most conclusive MVPA results). Some have suggested that such frontal activations recorded during the processing of incongruent scenes are task-dependent^24^. Arguably, these activations could arise because subjects need to inhibit the competing response induced by the scene’s gist (indeed, right inferior frontal cortex activations are found in response inhibition tasks ^80–82^), need to exert higher cognitive control^83^, or engage in online monitoring processes^84^. Yet in our experiment, these explanations are less plausible, since subjects were not asked to give any content-related responses, but simply to freely view the stimulus sequence and indicate how visible it was. In this passive viewing condition, we still found frontal activations - mainly in the inferior and middle frontal gyri (IFG and MFG, respectively) - bilaterally, while response inhibition is held to be mediated by the right IFG^82^. Our results accord with previous studies which implicated these areas in different types of associative processing, for related and unrelated objects^22^, for objects which have strong contextual associations^21^, for related and unrelated words^27^ and for sentence endings that were either unrelated^85,86^ or defied world-knowledge expectations^87^. In addition, increased mPFC connectivity with sensory areas has been found for congruent scenes which were better remembered than incongruent ones^28^, suggesting that this area mediates contextual facilitation of congruent object processing, and may be involved in extracting regularities across episodic experiences^88,89^.

Taken together, our findings seem to support the model suggested by Bar (2004) for scene processing. According to this model, during normal scene perception the visual cortex projects a blurred, low spatial-frequency representation early and rapidly to the prefrontal cortex (PFC; note that the model remains general about the exact areas within the PFC which are involved in the process, and that we specifically found inferior and medial frontal activations) and parahippocampal cortex (PHC). This projection is considerably faster than the detailed parvocellular analysis, and presumably takes place in the magnocellular pathway^90,91^. In the PHC, this coarse information activates an experience-based prediction about the scene’s context or its gist. Indeed, the gist of visual scenes can be extracted even with very short presentation durations of 13 ms^92^ (see also^93^, though see^94^), and such fast processing is held to be based on global analysis of low-level features^29^. Then, this schema is projected to the inferior temporal cortex (ITC), where a set of schema-congruent representations is activated so that representations of objects that are more strongly related to the schema are more activated, hereby facilitating their future identification. In parallel, the upcoming visual information of the target object selected by foveal vision and attention activates information in the PFC that subsequently sensitizes the most likely candidate interpretations of that individual object, irrespective of context^31^. In the ITC, the intersection between the schema-congruent representations and the candidate interpretations of the target object results in the reliable selection of a single identity. For example, if the most likely object representations in PFC include a computer, a television set, and a microwave, and the most likely contextual representation in the PHC correspond to informatics, the computer alternative is selected in the ITC, and all other candidates are suppressed. Then, further inspection allows refinement of this identity (for example, from a “computer” to a “laptop”).

Based on this model, we can now speculate about the mechanisms of incongruent scenes processing: in this case, the process should be prolonged and require additional analysis - as implied by the elevated activations in all three areas in our study in the incongruent condition. There, since the visual properties of the incongruent object generate different guesses about object-identities in a network of frontal areas than the schema-congruent representations subsequently activated in the ITC, the intersection fails to yield an identification of the object, requiring further inspection and a re-evaluation of both the extracted gist (PHC) and the possible object-guesses. This further inspection might lead to attentional engagement, as might be implied by the increased activations in the right superior parietal lobule^95^. Indeed, we previously showed that while attention is not captured by incongruent scenes, it is nevertheless engaged by them^58^. The difficulty to reach a match between the upcoming visual information and the activated guesses can explain the widely-reported disadvantage in identifying incongruent objects, both with respect to accuracy^6–8^ and to reaction times^3–5^. It is further strengthened by EEG findings, showing that the waveforms induced by congruent and incongruent scenes start to diverge in the N300 time window (200–300 ms after the scene had been presented) - if not earlier^96^, the time at which these matching procedures presumably take place, prior to object identification^9,16,18^ (though see^20^ and^19^). Note however, that this model holds the ITC as the locus of object-context integration, at which the information from the PHC and frontal areas converge. It also focuses on the perceptual aspect of object-context integration, to explain how scene gist affects object identification. Yet frontal activations (more specifically, IFG activations) were found also for verbal stimuli that were either semantically anomalous or defied world-knowledge expectations^97^, suggesting that (a) these frontal areas may also be involved in the integrative process itself, rather than only in generating possible guesses about object identities irrespective of context and (b) that they may mediate a more general, amodal mechanism of integration and evaluation.

### The role of consciousness in object-context relations processing

In this study, we found no neural evidence for processing of object-context relations in the absence of awareness. Using only trials in which subjects reported seeing nothing or only a glimpse of the stimulus, and were at chance in discriminating target image congruency (though slightly above chance in discriminating target image orientation), the BOLD activations found in the visible and unmasked conditions became undetectable in our setup. Following previous studies which failed to find univariate effects during unconscious processing but managed to show significant decoding^72^ we used multivariate pattern analysis (MVPA^98^) as a more sensitive way to detect neural activations which may subserve unconscious object-context integration; however, we did not find significant decoding in the invisible condition.

How should this null result be interpreted? One possibility is that it reflects the brain’s inability to process the relations between an object and a scene when both are invisible. This finding goes against our original behavioral finding of differential suppression durations for congruent vs. incongruent scenes^44^,see also^45^; however, a recent study failed to replicate^49^ this original finding, and we were also not able to replicate behavioral evidence for unconscious scene-object integration^50^. Furthermore, another study which focused on the processing of implied motion in real-life scenes also failed to find evidence of unconscious processing^99^. In the same vein, the findings of another study which investigated high-level contextual unconscious integration of words into congruent and incongruent sentences^100^ was recently criticized^101,102^ (for a review of studies showing different types of unconscious integration, see^48^). Taken together with the null result in our study, this could imply that high-level integration may actually not be possible in the well-controlled absence of awareness. This interpretation is in line with the prominent theories of consciousness which consider consciousness and integration to be intimately related^51,52^, if not equivalent to each other^53,54^.

On the other hand, one should be cautious in interpreting the absence of evidence as evidence of absence. The observable correlates of unconscious object-scene relations processing may be so weak that we missed them; our study was likely underpowered both with respect to number of subjects and number of trials, and fMRI may simply not be a sensitive enough methodology to detect the weak effects of unconscious higher-level processing. The Bayesian analyses we performed suggest that our data in the invisible condition was indeed inconclusive, and did not support the null hypothesis. Many previous fMRI studies either showed substantially reduced or no activations to invisible stimuli^103–105^, or effects that were significant, yet weaker and more focused compared with conscious processing e.g.^106–108^. Together, this raises the possibility that in some cases behavioral measures may be more sensitive than imaging results; in a recent study, for example, invisible reward cues improved subjects’ performance to the same extent as visible ones - yet while the latter evoked activations in several brain regions (namely, motor and premotor cortex and inferior parietal lobe), the former did not^109^. Critically, in that study subjects were performing a task while scanned. Our study, on the other hand, included no task in order to make sure that the observed activations were not task-induced, but rather represented object-context relations processing per se. Thus, while our findings cannot rule out the possibility that such processing indeed does not depend on conscious perception; they surely do not support this claim.

## Conclusions

While finding no evidence for unconscious processing of object-context relations, the present study contributes to our understanding of the underlying mechanisms during conscious processing. We found enhanced LOC, ITC, PHC and frontal (MFG, IFG, Cingulate) activations for incongruent scenes, irrespective of task requirements. Our results cannot be explained by low-level differences between the images, including spatial frequencies, which were suggested as a possible confound in previous studies^24^. The use of stimuli that depict people performing real-life actions with different objects, rather than non-ecological stimuli (e.g., line drawings^75^; isolated, floating objects^22,23^), or stimuli with which subjects have less hands-on, everyday experience (animals or objects presented in natural vs. urban sceneries^24–26^), enables us to better track real-life object-context relations processing which also occurs outside the laboratory. Arguably, in such real-life processes, incoming visual information about object features is compared with scene-congruent representations evoked by the scene gist, in an interplay between the abovementioned areas. This interplay - usually leading to contextual facilitation of object processing - is disrupted when incongruent scenes are presented, resulting in additional neural processing to resolve these incongruencies. Thus, our results go beyond previous studies by overcoming potential design limitations and providing further evidence for frontotemporal mechanisms of object-scene relations processing.

## Supplementary information

Supplementary file S1
