# Supplementary material for: Imaging object-scene relations processing in visible and invisible natural scenes
Source: Sci Rep. 2019 Mar 14;9:4567. doi: 10.1038/s41598-019-38654-z (PMC6418099; doi:10.1038/s41598-019-38654-z)
Supplement: Supplementary file 1 — Supplementary file S1 [file 41598_2019_38654_MOESM1_ESM.doc]

**Supplementary material**


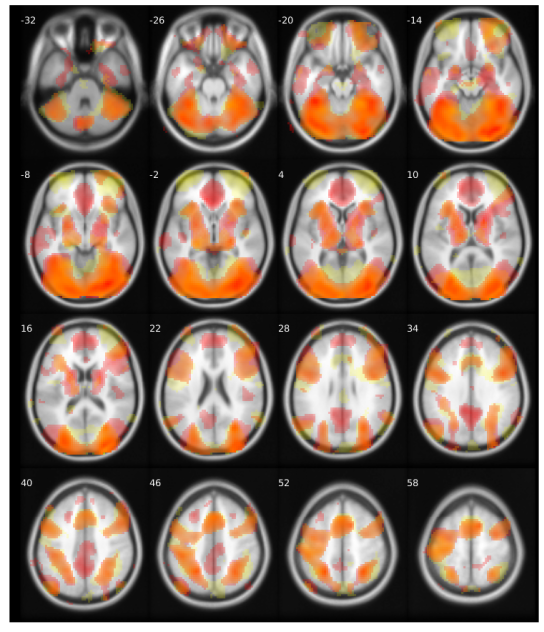


Supplementary figure 1. Comparison of baseline vs. stimulation (congruent and incongruent) trials in the visible condition (in red), and invisible condition (in yellow). Only voxels with uncorrected p-values < 0.001 are shown.
